# Supplementary material for: Rad27 and Exo1 function in different excision pathways for mismatch repair in Saccharomyces cerevisiae
Source: Nat Commun. 2021 Sep 22;12:5568. doi: 10.1038/s41467-021-25866-z (PMC8458276; doi:10.1038/s41467-021-25866-z)
Supplement: Supplementary file 1 — Supplementary Information [file 41467_2021_25866_MOESM1_ESM.pdf]

SUPPLEMENTARY INFORMATION

Rad27 and Exo1 function in different excision pathways for Mismatch Repair in *Saccharomyces cerevisiae*

By

Felipe A. Calil[1], Bin-Zhong Li[1], Kendall A. Torres[1], Katarina Nguyen[1], Nikki Bowen[1], Christopher D. Putnam[1, 2], and Richard D. Kolodner[1, 3, 4, 5]

From

Ludwig Institute for Cancer Research[1], Departments of Medicine[2] and Cellular and Molecular Medicine[3], Moores-UCSD Cancer Center[4] and Institute of Genomic Medicine[5], University of California School of Medicine, San Diego, 9500 Gilman Drive, La Jolla, CA 92093-0669

Address correspondence to:
Richard D. Kolodner
rkolodner@health.ucsd.edu
(858) 534-7804 (phone)
(858) 822-4479 (fax)

Running Title: Rad27 mediates excision during mismatch repair.

Supplementary Table 1. Significance of the mutation rate differences* between single and double mutant strains.

	exo1-Δ440-702 pms1-A99V	*exo1-Δ440-702 rad27Δ*	*pms1-A99V rad27Δ*
exo1-Δ440-702	$1.8x10^{-7}/1.8x10^{-7}$	$2.1x10^{-10}/5.2x10^{-16}$	-
pms1-A99V	$5x10^{-8}/7.4x10^{-6}$	-	$6.3x10^{-8}/3x10^{-12}$
rad27Δ	-	$5.4x10^{-5}/1.9x10^{-7}$	$1x10^{-10}/2.2x10^{-13}$

*Number at left is the p-value (two-sided Mann-Whitney U-test) for the *hom3-10* reversion assay. Number at right is the p-value for the *lys2-10A* reversion assay.

Supplementary Table 2. Significance of the mutation rate differences* between double and triple mutant strains.

	exo1-Δ440-702 pms1-A99V rad27Δ
exo1-Δ440-702 pms1-A99V	$3.2x10^{-7}/3.4x10^{-4}$
exo1-Δ440-702 rad27Δ	$9.9x10^{-9}/3.5x10^{-6}$
pms1-A99V rad27Δ	$1.7x10^{-3}/4.6x10^{-3}$

*Number at left is the p-value (two-sided Mann-Whitney U-test) for the *hom3-10* reversion assay. Number at right is the p-value for the *lys2-10A* reversion assay.

Supplementary Table 3. ***Saccharomyces cerevisiae*** **strains used in this study.**

Name	***Genotype***	***Reference***
RDKY5964	**MATa** *ura3-52 leu2Δ1 trpΔ63 his3Δ200 hom3-10 lys2::InsE-A10*	Reference 1
RDKY9658	RDKY5964 *msh2Δ::HIS3*	This study
RDKY9673	RDKY5964 *pms1Δ::HIS3*	This study
RDKY9796	RDKY5964 *rad27Δ::HIS3*	This study
RDKY4190	RDKY5964 *pms1-A99V*	Reference 2
RDKY9806	RDKY5964 *pms1-A99V rad27Δ::HIS3*	This study
RDKY10014	RDKY5964 *pms1-A99V exo1-Δ440-702::kanMX4*	This study
RDKY10017	RDKY5964 *pms1-A99V exo1-Δ440-702::kanMX4 rad27Δ::HIS3*	This study
RDKY9798	RDKY5964 *exo1-Δ440-702::kanMX4*	This study
RDKY9800	RDKY5964 *exo1-Δ440-702::kanMX4 rad27Δ::HIS3*	This study
RDKY10075	RDKY5964 *exo1-Δ440-702::kanMX4 rad27Δ::HIS3 pms1Δ::NAT*	This study
RDKY10073	RDKY5964 *exo1-Δ440-702::kanMX4 rad27Δ::HIS3 msh2Δ::NAT*	This study
RDKY8075	RDKY5964 *pol30-K217E*	Reference 3
RDKY9804	RDKY5964 *pol30-K217E rad27Δ::HIS3*	This study
RDKY7588	RDKY5964 *PMS1-4GFP::kanMX6*	Reference 1
RDKY7544	RDKY5964 *PMS1-4GFP::kanMX6 exo1Δ::hphNT1*	Reference 1
RDKY9783	RDKY5964 *PMS1-4GFP::kanMX6 exo1-Δ440-702::hphNT1*	This study
RDKY9785	RDKY5964 *PMS1-4GFP::kanMX6 rad27::HIS3*	This study
RDKY9787	RDKY5964 *PMS1-4GFP::kanMX6 exo1-Δ440-702::hphNT1 rad27Δ::HIS3*	This study
RDKY10020	RDKY5964 *PMS1-4GFP::kanMX6 exo1-Δ440-702::hphNT1 msh2Δ::NAT*	This study
RDKY10022	RDKY5964 *PMS1-4GFP::kanMX6 rad27Δ::HIS3 msh2Δ::NAT*	This study
RDKY10024	RDKY5964 *PMS1-4GFP::kanMX6 exo1-Δ440-702::hphNT1 rad27Δ::HIS3 msh2Δ::NAT*	This study

Supplementary Table 4. Primers used in this study.

Name	*Sequence*
Primer 1	5'-CAA TTA AGC GTA GGA AAT TAA GTA ATG CCA ATG TAG TCC AAG AAA CG TAG ATG AAT AAC GTA CGC TGC AGG TCG AC-3'
Primer 2	5'-TTT ACT GGG CAT TGA TTT TTT AAT TCT TGT CTT GAG GCA TTT CGA CGA GAT ATC GAT GAA TTC GAG CTC G-3'
Primer 3	5'-GGT CTA GTA CAA TGG CTT TTT CCC AAA GTA GAA GGC TTC TTA CTC CAA CCG TAC CCT GCG TAC GCT GCA GGT CGA C-3'
Primer 4	5'-GAC AAT GGC AAT TAA GCG TAG GAA ATT AAG TAA TGC CAA TGT AGT CCA AGA AAC GTA GAT GAA TAA ATC GAT GAA TTC GAG CTC G-3'
Primer 5	5'-TAT GCC AAG GTG AAG GAC CAA AAG AAG AAA GTG GAA AAA GAA CCC CCT CAT CCT GAT GCG GTA TTT TCT CC-3'
Primer 6	5'-CAG CAT ACA TTG GAA AGA AAT AGG AAA CGG ACA CCG GAA GAA AAA ATA TGC GTT TCG GTG ATG ACG GTG-3'
Primer 7	5'-AGT TGT TTG TTG ATG ACT GC-3'
Primer 8	5'-TTC AGA AGC TTC TTC TGG AG-3'
Primer 9	5'-CTT TCC TGG TTC AAG CAT TG-3'

a

	mutation spectra						7T>6T mutation rate		
	ΔC	ΔT	ΔG	ΔT	ΔG	ΔT			
wild type	0	0	**1**	**31**	0	**1**	1.66	[0.7 - 4.1]	x 10^{-9}
msh2Δ	0	0	0	**36**	0	0	3240.	[2600 - 4330]	x 10^{-9}
exo1Δ440-702	**1**	0	**1**	**24**	**1**	0	4.74	[3.9 - 5.8]	x 10^{-9}
rad27Δ	**1**	0	**1**	**22**	0	0	157.	[87 - 310]	x 10^{-9}
rad27Δ exo1Δ440-702	0	**1**	0	**26**	0	0	540.	[324 - 723]	x 10^{-9}

GT**C**TT**T**ACAGG**G**TTTTTT**T**G**G**TT**T**AG

b

Variant	Sequence	Translation
HOM3	GTCTTTACAGGGTTTTTT-GGTTTAG<br>ValPheThrGlyPhePhe-GlyLeu	VFTGFFGL
hom3-10	GTCTTTACAGGGTTTTTTTGGTTTAG<br>ValPheThrGlyPhePhe**TrpPhe**…	VFTGFF**WF**...
ΔC revertant	GT-TTTACAGGGTTTTTTTGGTTTAG<br>Va-l**LeuGln**GlyPhePheGlyLeu	V**LQ**GFFGL
ΔT revertant	GTCTT-ACAGGGTTTTTTTGGTTTAG<br>Val**Le-uGln**GlyPhePheGlyLeu	V**LQ**GFFGL
ΔG revertant	GTCTTTACAGG-TTTTTTTGGTTTAG<br>ValPheThrGl-yPhePheGlyLeu	VFTGFFGL
ΔT revertant	GTCTTTACAGGGTTTTTT-GGTTTAG<br>ValPheThrGlyPhePhe-GlyLeu	VFTGFFGL
ΔG revertant	GTCTTTACAGGGTTTTTTTG-TTTAG<br>ValPheThrGlyPhePhe**Cy-s**Leu	VFTGFF**C**L
ΔT revertant	GTCTTTACAGGGTTTTTTTGTTT-AG<br>ValPheThrGlyPhePhe**Cys**Le-u	VFTGFF**C**L

Supplementary Figure 1. Reverting mutations recovered using the *hom3-10* frameshift assay. a The number of each mutation that revert the *hom3-10* 7T frameshift (underlined) recovered (indicated as a deletion at bottom) from each genotype are displayed. The 7T>6T mutation rates calculated based on the total reversion rate and the frequency of 7T>6T reversions observed are listed at right. **b** The effect of the various single base pair deletion revertants observed in panel A on the protein sequence of Hom3. Changes from the wild-type sequence are displayed in bold.

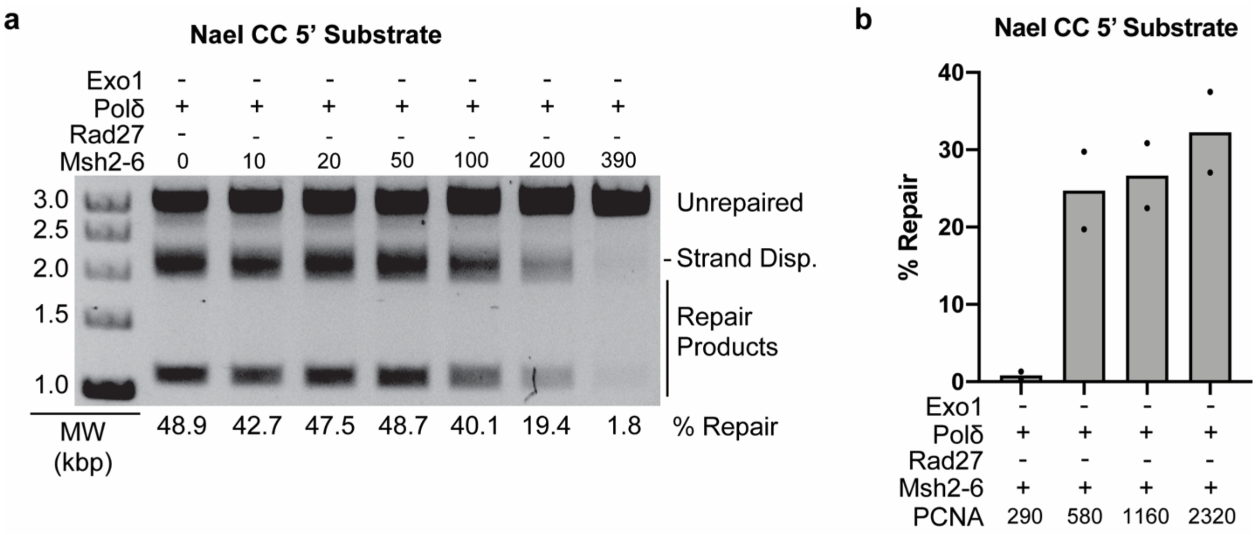

Supplementary Figure 2. Msh2-Msh6 and PCNA titrations eliminate suppression of strand-displacement synthesis. a-b Assays of 5′ nick-directed repair of the CC substrate, in which different proteins were omitted or substituted as indicated. All reactions also contain PCNA, RFC-Δ1N, and RPA. Other than Msh2-Msh6 and PCNA (fmole), the amounts of all proteins used in the assays are as listed in the Methods section. MW, molecular weight markers. The percent repair corresponds to the fraction of all DNA in each individual lane susceptible to PstI cleavage, including those labeled "strand displacement" and "repair products". **b** The amount of repair for the reactions was quantified. Two independent experiments were performed, the average amount of repair observed is reported and individual values from the different experiments are indicated by the dots on each histogram bar. Bar graph was constructed in GraphPad Prism software.

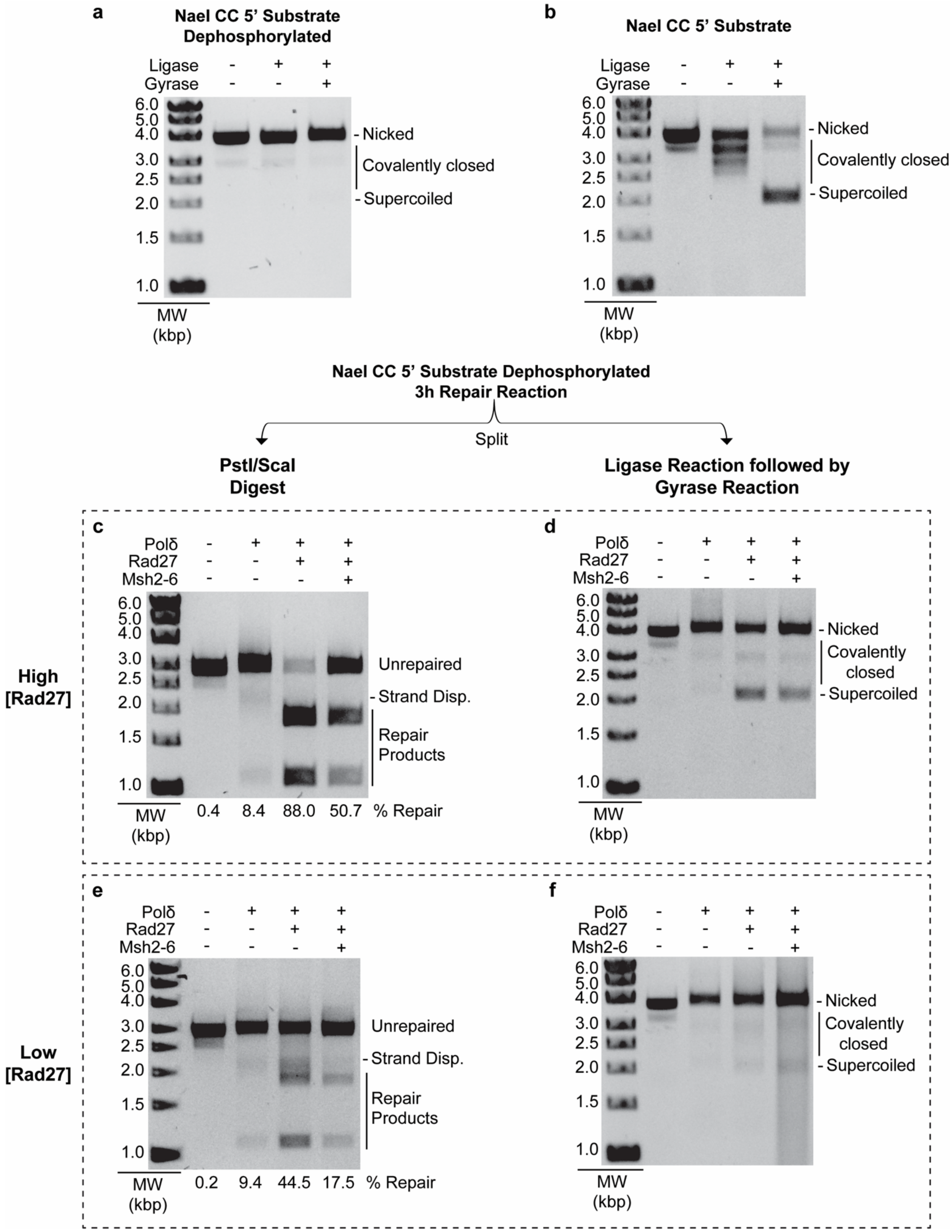
a
Nael CC 5' Substrate Dephosphorylated
Ligase - + +
Gyrase - - +
6.0 5.0 4.0 3.0 2.5 2.0 1.5 1.0
MW (kbp)
Nicked
Covalently closed
Supercoiled
b
Nael CC 5' Substrate
Ligase - + +
Gyrase - - +
Nicked
Covalently closed
Supercoiled
MW (kbp)
Nael CC 5' Substrate Dephosphorylated
3h Repair Reaction
Split
PstI/ScaI Digest
Ligase Reaction followed by Gyrase Reaction
High [Rad27]
c
Polδ - + + +
Rad27 - - + +
Msh2-6 - - - +
Unrepaired
Strand Disp.
Repair Products
MW (kbp)
0.4 8.4 88.0 50.7 % Repair
d
Polδ - + + +
Rad27 - - + +
Msh2-6 - - - +
Nicked
Covalently closed
Supercoiled
MW (kbp)
Low [Rad27]
e
Polδ - + + +
Rad27 - - + +
Msh2-6 - - - +
Unrepaired
Strand Disp.
Repair Products
MW (kbp)
0.2 9.4 44.5 17.5 % Repair
f
Polδ - + + +
Rad27 - - + +
Msh2-6 - - - +
Nicked
Covalently closed
Supercoiled
MW (kbp)

Supplementary Figure 3. Rad27-cleaved mature products can be ligated and supercoiled. **a** Control experiment with dephosphorylated 5′ nicked CC substrate, which is resistant to ligation by T4 DNA ligase and subsequent supercoiling by DNA gyrase. **b** Control experiment with 5′ nicked CC substrate, which is ligated by T4 DNA ligase and subsequently supercoiled by DNA gyrase. **c-f** Representative repair assays with dephosphorylated 5′ nick-directed repair of the CC substrate dephosphorylated, in which different proteins were omitted or substituted as indicated. All reactions also contain PCNA, RFC-Δ1N, and RPA. The DNA present in the reactions was purified and analyzed for repair by digestion with PstI and ScaI (**c,e**) or analyzed for the presence of ligatable nicks by treatment with T4 DNA ligase and subsequently with DNA gyrase reactions (**d,f**). Two different amounts of Rad27 were tested: 5 pmole (**c,d**) and 2.5 pmole (**e,f**). MW, molecular weight markers. The percent repair corresponds to the fraction of all DNA in each individual lane susceptible to PstI cleavage, including those labeled “strand displacement” and “repair products”. **a-f** A minimum of two independent experiments were performed.

Supplementary References

1. Hombauer, H., Campbell, C. S., Smith, C. E., Desai, A. & Kolodner, R. D. Visualization of eukaryotic DNA mismatch repair reveals distinct recognition and repair intermediates. *Cell* **147**, 1040-1053 (2011).
2. Amin, N. S., Nguyen, M. N., Oh, S. & Kolodner, R. D. exo1-Dependent mutator mutations: model system for studying functional interactions in mismatch repair. *Molecular and cellular biology* **21**, 5142-5155 (2001).
3. Goellner, E. M. *et al.* Identification of Exo1-Msh2 interaction motifs in DNA mismatch repair and new Msh2-binding partners. *Nature structural & molecular biology* **25**, 650-659, doi:10.1038/s41594-018-0092-y (2018).
