# Supplementary material for: Rad27 and Exo1 function in different excision pathways for mismatch repair in Saccharomyces cerevisiae
Source: Nat Commun. 2021 Sep 22;12:5568. doi: 10.1038/s41467-021-25866-z (PMC8458276; doi:10.1038/s41467-021-25866-z)
Supplement: Supplementary file 3 — Reporting Summary [file 41467_2021_25866_MOESM3_ESM.pdf]

nature research

Corresponding author(s): Richard Kolodner

Last updated by author(s): Aug 24, 2021

Reporting Summary

Nature Research wishes to improve the reproducibility of the work that we publish. This form provides structure for consistency and transparency in reporting. For further information on Nature Research policies, see our Editorial Policies and the Editorial Policy Checklist.

Statistics

For all statistical analyses, confirm that the following items are present in the figure legend, table legend, main text, or Methods section.

n/a	Confirmed	
☐	☒	The exact sample size (*n*) for each experimental group/condition, given as a discrete number and unit of measurement
☐	☒	A statement on whether measurements were taken from distinct samples or whether the same sample was measured repeatedly
☐	☒	The statistical test(s) used AND whether they are one- or two-sided<br>*Only common tests should be described solely by name; describe more complex techniques in the Methods section.*
☒	☐	A description of all covariates tested
☒	☐	A description of any assumptions or corrections, such as tests of normality and adjustment for multiple comparisons
☐	☒	A full description of the statistical parameters including central tendency (e.g. means) or other basic estimates (e.g. regression coefficient) AND variation (e.g. standard deviation) or associated estimates of uncertainty (e.g. confidence intervals)
☐	☒	For null hypothesis testing, the test statistic (e.g. *F*, *t*, *r*) with confidence intervals, effect sizes, degrees of freedom and *P* value noted<br>*Give P values as exact values whenever suitable.*
☒	☐	For Bayesian analysis, information on the choice of priors and Markov chain Monte Carlo settings
☒	☐	For hierarchical and complex designs, identification of the appropriate level for tests and full reporting of outcomes
☒	☐	Estimates of effect sizes (e.g. Cohen's *d*, Pearson's *r*), indicating how they were calculated

Our web collection on statistics for biologists contains articles on many of the points above.

Software and code

Policy information about availability of computer code

Data collection	AlphaView version 3.4.0<br>ECHO Pro version 6.0
Data analysis	Microsoft Excel version 16.43<br>Graphpad Prism version 8.3.0<br>Adobe Photoshop CS6

For manuscripts utilizing custom algorithms or software that are central to the research but not yet described in published literature, software must be made available to editors and reviewers. We strongly encourage code deposition in a community repository (e.g. GitHub). See the Nature Research guidelines for submitting code & software for further information.

Data

Policy information about availability of data

All manuscripts must include a data availability statement. This statement should provide the following information, where applicable:
- Accession codes, unique identifiers, or web links for publicly available datasets
- A list of figures that have associated raw data
- A description of any restrictions on data availability

All data generated by this study are provided in either the main text and figures or the accompanying Supplementary Information or Supplementary Source Data.

Field-specific reporting

Please select the one below that is the best fit for your research. If you are not sure, read the appropriate sections before making your selection.

[x] Life sciences [ ] Behavioural & social sciences [ ] Ecological, evolutionary & environmental sciences

For a reference copy of the document with all sections, see nature.com/documents/nr-reporting-summary-flat.pdf

Life sciences study design

All studies must disclose on these points even when the disclosure is negative.

Sample size	Sample size for fluctuation analysis of mutation rates were a minimum of 2 independently derived strains and 14 or more cultures based on what has been used in numerous previous studies (PMID: 11438669, 22118461). Statistical significance was determined by 95% confidence intervals for large differences and by Mann-Whitney two-sided tests for small differences. 14 cultures results in 95% confidence intervals that exclude outliers.<br>Sample size for microscopy experiments were based on what has been used in numerous previous studies (PMID: 22118461, 24811092).
Data exclusions	No data were excluded from the analyses.
Replication	The experiments were repeated a minimum of 2-3 times, as indicated, independently to confirm reproducibility. All experiments were replicated successfully.
Randomization	No randomization was performed in this paper because no mammalian cell lines, animal or human subjects are involved. Mutant yeast strains were compared to isogenic wildtype yeast strains.
Blinding	Blinding was not performed during data collection or analysis. This is because all data were collected by machines and analysis were performed by using programs and protocols which treat all samples equally, so it is unlikely that bias can be introduced. For all genetic experiments, data analysis was performed by a person who was not involved in collecting the data.

Reporting for specific materials, systems and methods

We require information from authors about some types of materials, experimental systems and methods used in many studies. Here, indicate whether each material, system or method listed is relevant to your study. If you are not sure if a list item applies to your research, read the appropriate section before selecting a response.

Materials & experimental systems

n/a	Involved in the study	
[x]	[ ]	Antibodies
[ ]	[x]	Eukaryotic cell lines
[x]	[ ]	Palaeontology and archaeology
[x]	[ ]	Animals and other organisms
[x]	[ ]	Human research participants
[x]	[ ]	Clinical data
[x]	[ ]	Dual use research of concern

Methods

n/a	Involved in the study	
[x]	[ ]	ChIP-seq
[x]	[ ]	Flow cytometry
[x]	[ ]	MRI-based neuroimaging

Eukaryotic cell lines

Policy information about cell lines

Cell line source(s)	Yeast strains from the RDKY laboratory collection. All strains are derived from the S288C strain background.
Authentication	No mammalian cell lines were used. Gene knockouts in yeast strains were confirmed by PCR and sequencing.
Mycoplasma contamination	Mycoplasma do not grow in yeast cell cultures. No mammalian cell cultures were used.
Commonly misidentified lines (See ICLAC register)	No commonly misidentified cell lines were used.
