# Supplementary figures and images for: Rad27 and Exo1 function in different excision pathways for mismatch repair in Saccharomyces cerevisiae
Source: Nat Commun. 2021 Sep 22;12:5568. doi: 10.1038/s41467-021-25866-z (PMC8458276; doi:10.1038/s41467-021-25866-z)

## SUPPLEMENTARY DATA SET – UNCROPPED GELS

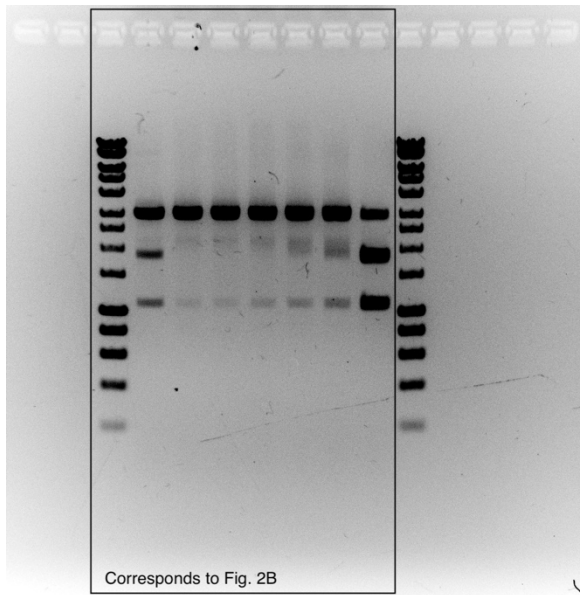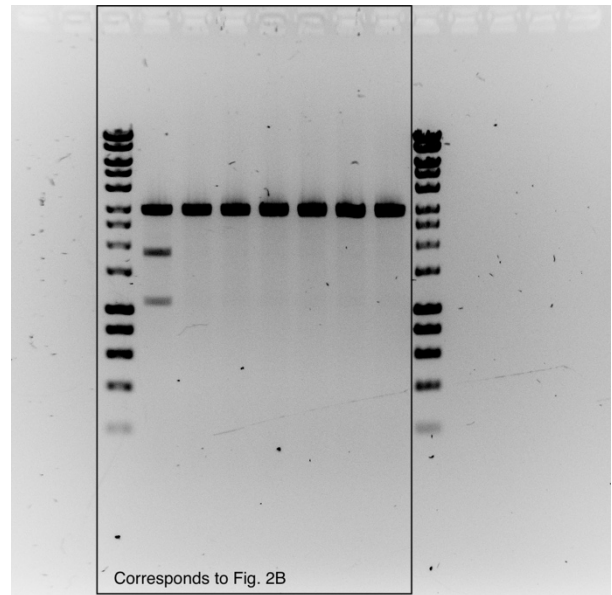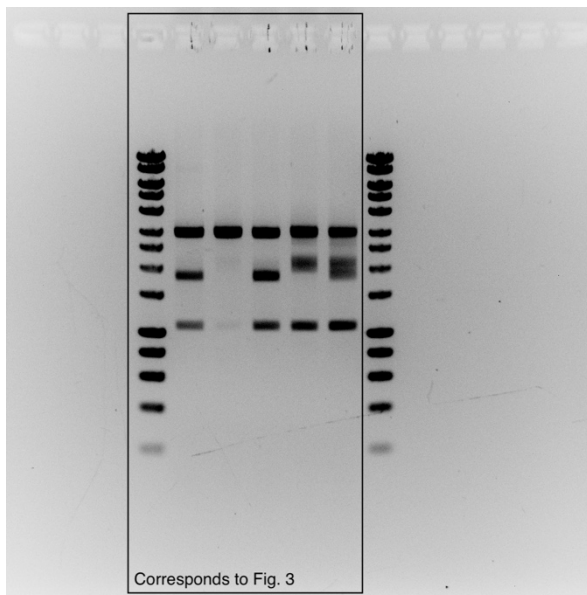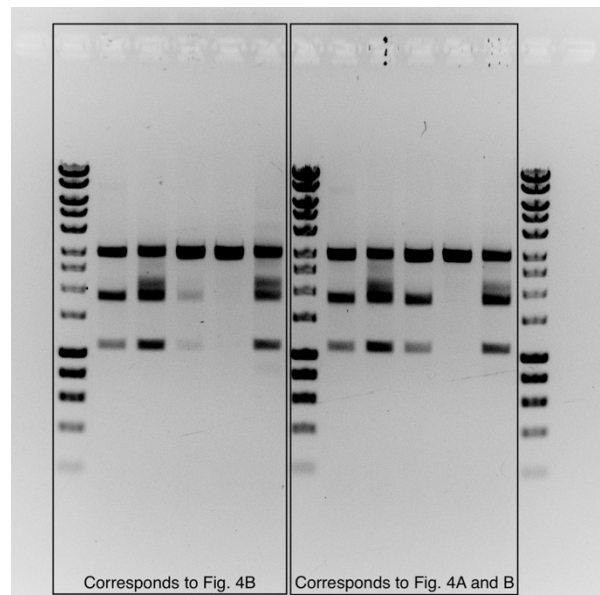

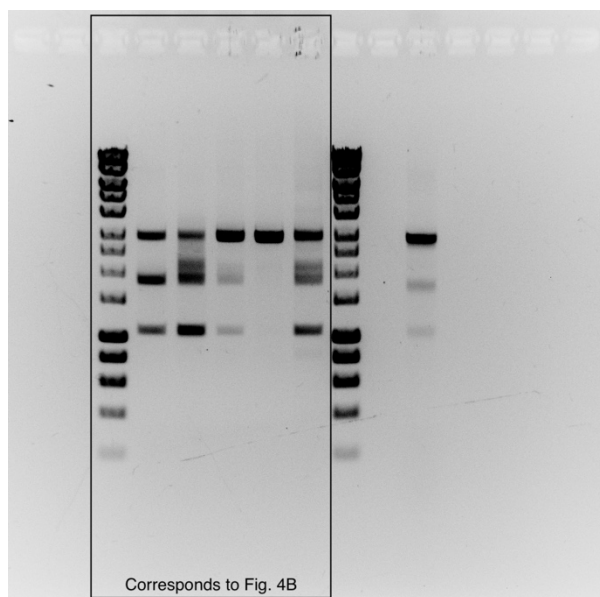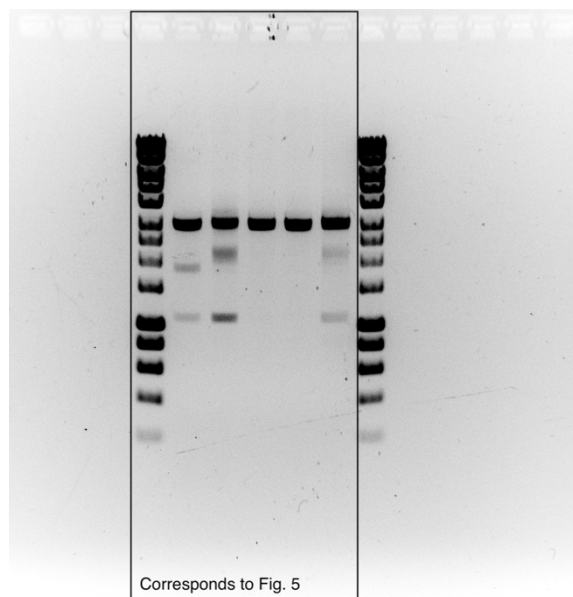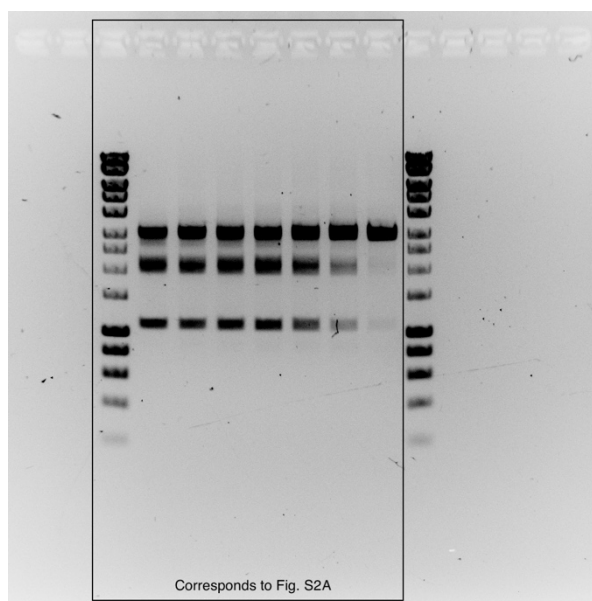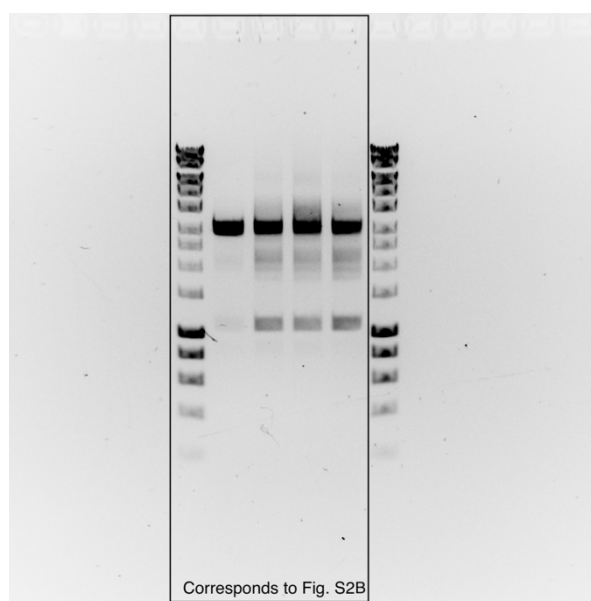

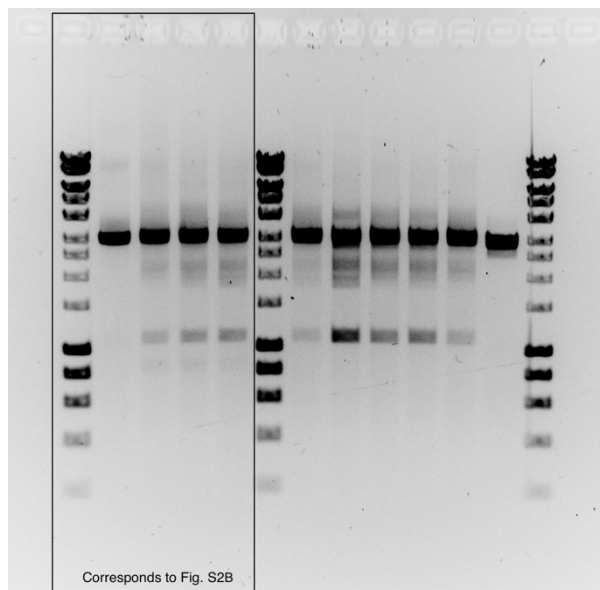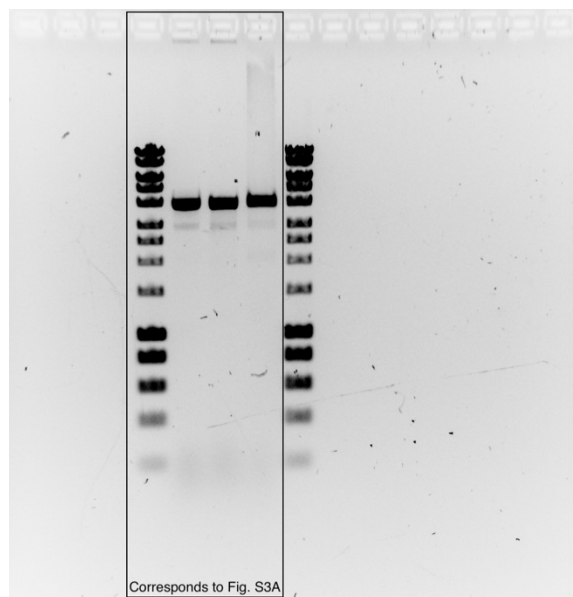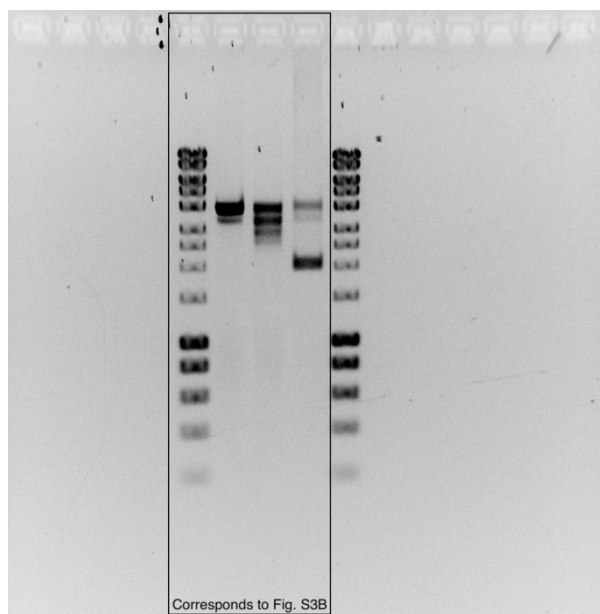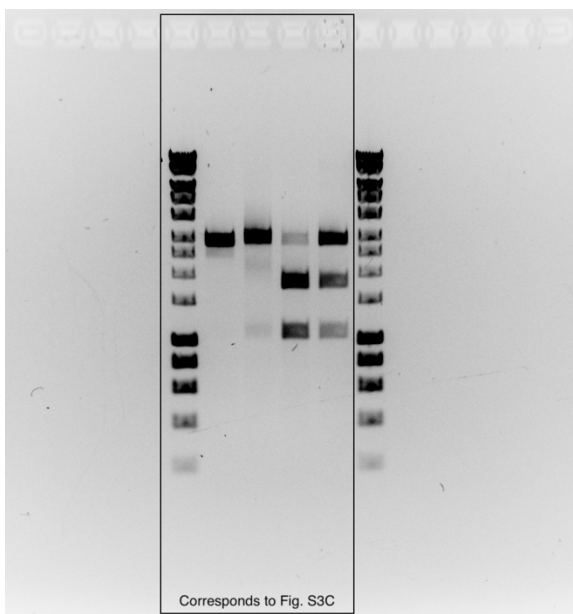

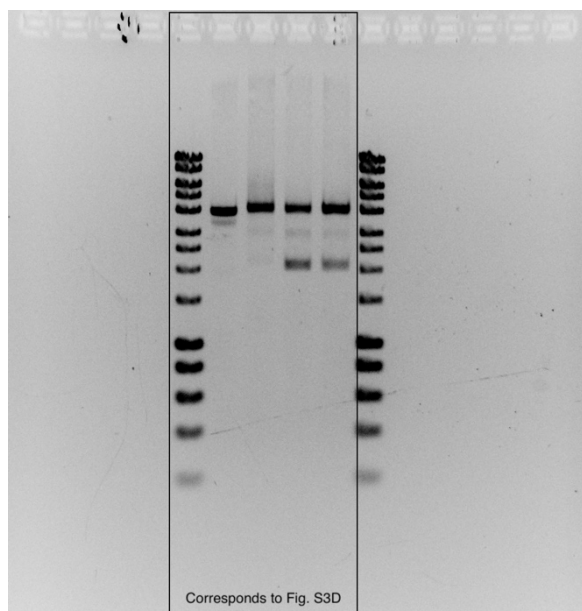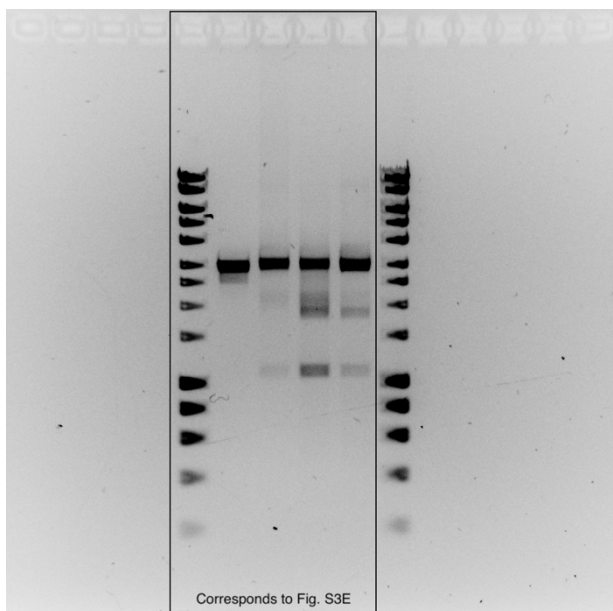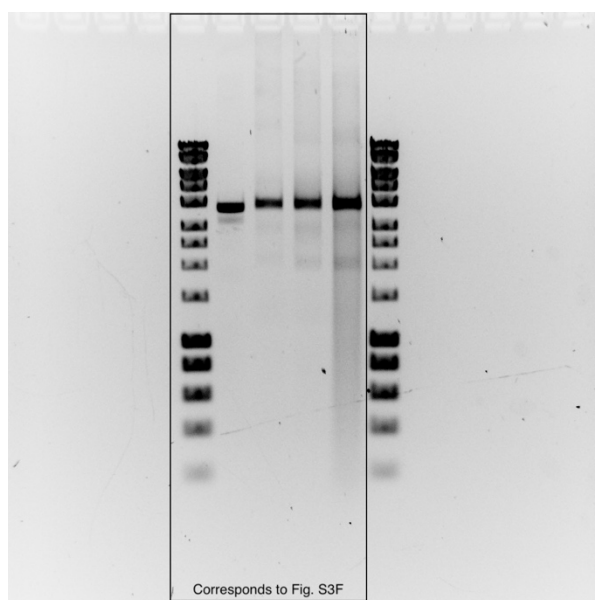

Supplement: Supplementary file 4 — Source Data [file 41467_2021_25866_MOESM4_ESM.pdf]
